# Prediction of Apple Hybrid Offspring Aroma Based on Hyperspectral

**DOI:** 10.3390/foods11233890

**Published:** 2022-12-02

**Authors:** Huili Zhu, Minyan Wang, Jing Zhang, Fengwang Ma

**Affiliations:** State Key Laboratory of Crop Stress Biology for Arid Areas, Shaanxi Key Laboratory of Apple, College of Horticulture, Northwest A&F University, Xianyang 712100, China

**Keywords:** apple, hyperspectral, hybrid offspring, random forest algorithm, HSI

## Abstract

Used Random forest algorithm to construct a prediction model of aroma components based on the hybrid offspring of ‘Honeycrisp’ × ‘Maodi’, and different preprocessing methods were tried (Standardization (SS), First-order Derivative (D1) and Standard normal variate (SNV)). The aroma composition and content were determined by gas chromatography-mass spectrometry (GC-MS), and the main aroma components of apples were classified according to compound categories, including ester, aldehyde, ketone, alcohol. Taking the chemical groups as the research objects, the characteristic wavelengths were selected by grid search algorithm, and the characteristic wavelength-aroma chemical group model was established, and the same method was used to construct the model for single aroma components. The results show: SNV has the best noise removal effect among the five preprocessing methods. Under the SNV treatment, aroma chemical groups of apples showed a good correlation with the spectrum. The number of characteristic spectra of ester are 413, 493, 512, 551, 592, 600, 721, 727, 729, 733 nm, all in the visible light range. The determination coefficient ($R^{2}$), the root mean square error (RMSE) and the ratio of the standard deviation values (RPD) of validation were 0.90, 4936.16 and 1.13. The characteristic spectrum of alcohols is 519, 562, 570, 571, 660, 676, 737, 738 nm, the range is close to that of ester. The $R^{2}$ and RMSE of alcohol validation are 0.92 and 83.21, and RPD is 1.30. The number of characteristic spectra of aldehyde is 20, and the most important band is 1000 nm, which is outside the visible light range. The number of characteristic spectra of ketone is 15, and also has some distribution outside the visible light range. The $R^{2}$ of aldehyde and ketone validation are 0.84 and 0.86. Except for cyclooctanol, the $R^{2}$ of single aroma compound prediction model performed poorly. Based on the models, we tried to visualize alcohol, which can roughly represent their distribution on apple. Their distributions all show significant differences in the center and edge of apple, but the results are still rough due to the accuracy of models. In conclusion, the study can preliminarily prove that hyperspectral imaging technology (HSI) can perform non-destructive detection of aroma in apple hybrid offspring.

## 1. Introduction

Apple is one of the most popular and valuable fruit in the world. Texture, flavor and nutrition are the most important and economically valuable qualities of apple [1]. The quality of apples mainly includes two aspects, physical properties represented by color, texture and size, and chemical properties composed of soluble solids, polyphenols, aroma, etc. Generally, aroma is believed to play a leading role in apple flavor [2]. Exploring the aroma composition can determine the quality and processing potential of apple. In addition, improving the aroma of apple has always been one of the goals of breeding efforts, and screening from numerous hybrid offspring during the breeding process requires a lot of aromatic testing. Thus, it is of great significance to construct an apple aroma composition assessment model.

Hyperspectral imaging technology (HSI) is developed from near-infrared spectropy (NIR), and the principle is that some chemical components have different ability to reflect light in different bands. Near-infrared spectroscopy has the disadvantage of only collecting specific site information, while hyperspectral technology combines two-dimnesional imaging technology to achieve spectral full-image information acquisition [3]. With the maturity of spectroscopic technology and the development of computer processing technology, hyperspectral imaging technology has been widely used in agriculture, food and chemical fields due to its fast and efficient and non-destructive characteristics [4]. Since hyperspectral data is a comprehensive reflection of the internal spatial structure and chemical composition of the sample, this detection technique has unique advantages in non-destructive testing applications of fruit [5]. Fernando completed the detection of three kinds of apple hardness and SSC in “Golden Delicious”, “Jonagold” and “Red Delicious”, using hyperspectral techniques and partial least squares regression models [6]. Ding Chengqiao used this method to complete the detection of minor damage to apple [7]. Feng Zipeng used spectroscopic technology to design an apple mold heart detection device [8]. Hyperspectral imaging also has applications in other fruits, such as kiwifruit [9], banana [10] and pear [11].

During the past decades, apple have been extensively bred and a large number of new cultivars have been developed [12]. Agronomic research has focused on parameters such as yield, post-harvest shelf-life, disease resistance and stress resistance, while sensory quality was pushed towards the background. As a result, cultivars with high-yield and superior fruit size replaced the tasteful old cultivars, which is called genetic erosion [13]. The emergence of the genetic erosion phenomenon is not only market-oriented, but also subject to the instant detection of aroma components and the level of sensory research of aroma sensory at level. With the further upgrading of consumption, the market has more stringent requirements for apple aroma, and it is the general trend that aroma is added to breeding plans of apple. However, prediction of aroma components by HSI, especially single aroma substance, has been mostly applied in tea, coffee and spices, etc.; and rarely used in the research of fruit aromas. Nicola Caporaso predicted single volatile compounds in single roasted coffee by HSI in the spectral region 1000–2500 nm [14]. This has not been tried in fruits yet, but Su Wenhao achieved a prediction for volatile chlorpyrifos (an organophosphate insecticide) in jujube [15]. Jin Xuemei constructed a prediction model of grape aroma categroies through using hyperspectral imaging technology in spectral region 400–1000 nm combined with PLS algorithm [16]. According to previous studies, most of the samples are from the single variety, which only meet the detection needs of market and processing. However, in the process of variety breeding, the aroma difference between the hybrid offspring is huge. The model constructed by a single variety is not competent, and detecting the hybrid offspring one by one is too expensive and cumbersome using trandtional method of gas chromatography-mass spectrometry (GC-MS) [17].

Consequently, the main aim of this study is to apply hyperspectral imaging to establish a predictive model of the aroma composition of hybrid offspring of ‘Honeycrisp’ × ‘Maodi’, and initially realize the instant detection of apple components, so as to provide a theoretical basis for high-efficiency and low-cost flavor breeding.

## 2. Materials and Methods

### 2.1. Apples Fruit

The experiment was conducted on 32 different hybrid offspring with different aromatic characteristics of “Honeycrisp” and “Maodi”. In 2021, all apples were harvested in the experimental orchard at Luo Chuan (Shaanxi, China). This orchard is located at $109^{{}^{\circ}}29^{\prime }$ east longitude, $35^{{}^{\circ}}16^{\prime }$ north latitude, 1072 m above sea level, which belongs to the warm temperate semi-humid continental monsoon climate, with an average annual temperature of 9.2 °C, 600 mm precipitation, 2552 h of sunshine, frost-free period 167 d. All apple were picked in three batches on 9 September, 17 September and 1 October, 2021. After the commercial harvesting (judged by the iodine liquid method), all apples were stored in a cold chamber at 4 °C and at around 90% of humidity until their characterization (October 2021).

### 2.2. Sample Preparation and Analysis of Volatile Aroma Compounds by GC-MS

5 apples of uniform appearance, without mechanical damage and disease-free were collected from each hybrid offspring, to form the experimental group. After acquired the hyperspectral images, fruit of the experimental group were powdered in liquid nitrogen, then stored at −80 °C for GC-MS analysis. The volatile compounds of each sample extracted by solid phase micro extraction (SPME). Approximately 5.0 g frozen fruit (power), 1.0 g NaCl and 10 μL 3-nonnanone (0.04 μL·mL${}^{-1}$) as an internal standard sealed in a 50 mL vial, then the headspace gases were extracted for 40 min at 40 °C using fiber coated with 50/30 μm divinylbenzene/carboxen/polydimethylsilozane (DVA/CAR/PDMS). Subsequently, sample were directly injected into the injection port of a GC-MS (QP2010 SE) with a splitless mode for 2.5 min at 230 °C. Volatile analysis was performed using a HP-INNOWax column (60 m × 0.25 mm inner diameter × 0.25 μm film thickness) with a carrier gas helium flow rate of 34 cm·s${}^{-1}$. The initial oven temperature was maintained at 40 °C for 3 min, followed by a temperature increase at a rate of 5 °C·min${}^{-1}$ to 150 °C, then to 220 °C at a rate of 10 °C·min${}^{-1}$ then maintained at 220 °C for 5 min. The energy of electron impact (EI) was 70 eV. The transfertemperature and sourcetemperature were 240 °C. Aroma volatiles were qualitatively analyzed by comparison with mass spectra (MS) from NIST 2017 Library. Aroma Volatile were relatively quantified using the peak area of the internal standard (3-nonanone) [18].

### 2.3. Hyperspectral Imaging (HSI) System

Images were captured with laboratory based 12 bit line scanner camera (Pika XC2, Bozeman, MT, USA) with a lens with 23 mm focal length and 4 current controlled wide-spectrum quartz-halogen lights. The camera had a spectral resolution of 1.3 nm and captured 462 wavelengths between 400 and 1000 nm. Apples were positioned on a black tray on a translation stage, the exposure time was adjusted to 19.4 ms, and the translation stage moved at 1.23 mm·s${}^{-1}$. Image acquisition and data extraction were conducted using software of Spectronon (Version 2.112; Resonon, Bozeman, MT, USA) and Python (Version 3.7.3). The mean raw reflectance of each image was extracted by marking a region of interest (ROI) for each sample. The ROI for images contained one side of apples excluding the area in the center of the fruit where reflection was very intense and the area in the edge of fruit where reflection was insufficient, only 15% of the part of the image was retained where reflection was moderate. The mean corrected relative reflectance (R) were calculated from the raw spectral reflectance, $R_{0}$, as shown in Equation (1):

(1) $$R=\frac{R_{0}-D}{W-D}$$

In the Equation, *D* is the reflectance of a dark image (camera lens covered) and *W* is the reflectance of a white Teflon sheet that reflects 99% of incident light. This corrects for the spectral curve of the fruit surface. The 100% reflectivity was scaled to 10,000 (integers) by default. The mean corrected relative reflectance was used for model development [19].

### 2.4. Imaging Pre-Processing and Feature Wavelength Selection

The original dataset with 160 images was partitioned into two independent part of calibration set and validation set (3:2) by train_test_split function of sklearn package. The pre-processing of the hyperspectral images and the selection of region of interest (ROI) were performed with python 3.7 software using the spectral and numpy packages. All selected spectra were gathered into a matrix X (32 hybrid offspring × 5 fruit). After pre-tests, ROIs was preprocessed with the three methods (Standardization, First-order Derivative and Standard normal variate) to increase its signal to noise ratio and compare the effects of methods [13].

Due to the high volume of data, it is not possible using a common computer to analysis all wavelength of images. The selection of feature wavelength is necessary. In order to select the optimal combination of parameters, this work used grid search algorithm with 10-fold cross-validation. Futhormore, in order to eliminate a subset of anomalous samples, 80% of apples were randomly sampled using Monte Carlo cross-validation method (MCCV) with a cycle of 1000 and the best combination of modeling was selected.

### 2.5. Modelling

After pre-treatment, the average spectra of ROIs were used for modelling with the relevant parameters. Random forest (RF) regression was applied to build models for determining apple aroma, using Python 3.7 software coupled with several packages include ‘numpy’, ‘pandas’, ‘scipy’, ‘math’, ‘matplotlib’ and ‘sklearn’.

The determination coefficient ($R^{2}$), the root mean square error (RMSE) and the ratio of the standard deviation values (RPD) were used to assess the developed model performance.

## 3. Results

### 3.1. Aroma Profile of Apples

**Figure 1:**
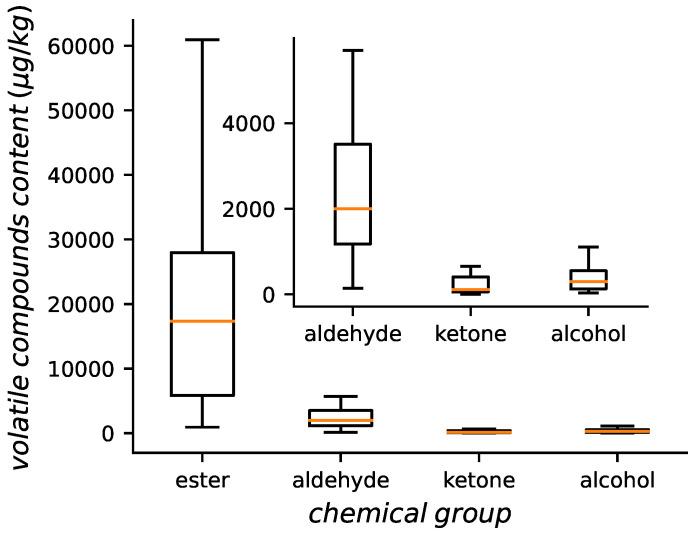
Aroma composition of apples.

Aroma is an essential sensorial property, which determined by the kinds and proportion of volatile compounds. In this work, a total of 153 compounds were identified. Ignoring some substances that were not easy to classify, esters were the most abundant (53), followed by alcohols (26), aldehydes (19), alkane (14), ketones (11). The type and content of the aroma is similar to previous reports [20]. In order to analyzed the overall apple profile, the total content of the above aroma categories is summarized (Figure 1).

### 3.2. Spectral Analysis and Preprocessing

**Figure 2:**
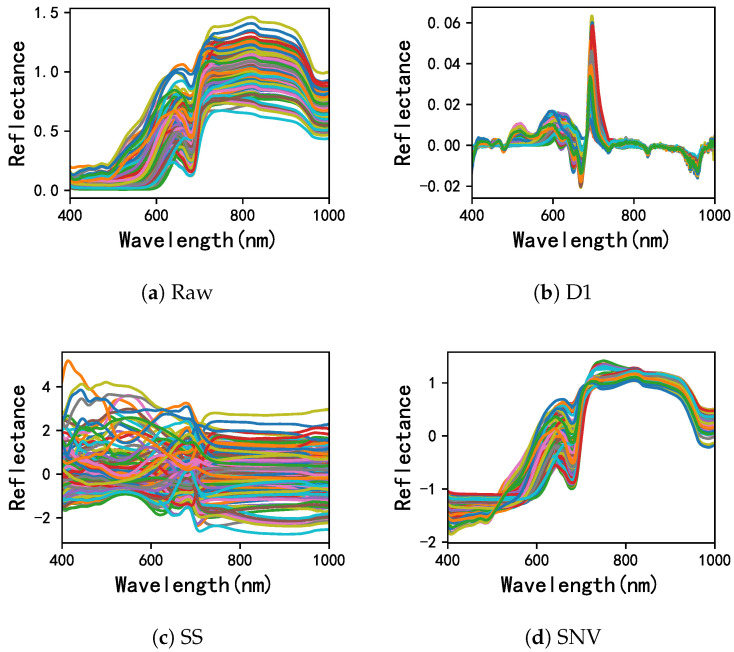
Spectra pre-treatment of HSI image.

The average spectral curve with standard deviation of fruits, ranging from 400 to 1000 nm, was shown as Figure 2a. It was found that all apples had the similar spectral trends. The difference in the visible region (400–750 nm) was attributed to varied color and the vacuoles and chloroplasts in the peel of apples [21]. The reflectance around 840 nm was assigned to the sugar content [22], and the 970 nm caused by the water content [23]. In addition, bands of 401,693,938 and 951 nm were used for non-destructive testing of grape aroma profile [16]. Moreover, the reflectance after preprocessing followed the same pattern as before and weakened the influence of ambient noise. The mainstream HSI preprocessing methods are Standardization (SS, Figure 2c), First-order Derivative (D1, Figure 2b) and Standard normal variate (SNV, Figure 2d). As shown in Figure 2, D1 and SNV had the best effect on the elimination of ambient noise and the best for the beam reduction of the sample spectral waveform, consistent with those of [24].

After preprocessing, build random forest (RF) model by correction set and perform a 10-fold cross-validation to obtain the optimal parameters, which are then modeled using validation set. As shown as Table 1, spectra pre-treatment effectively eliminated ambient noise, but also some valuable information.

### 3.3. Prediction of Chemical Groups of Apples

The aroma composition of the hybrid offspring depends on the genetic traits and cultivation, also vary individually on an apple to apple basis. However, the number of hybrid offspring is large and the complexity of aroma metabolism process, so it is almost impossible to track and measure the synthesis reaction process of multiple classes of aroma simultaneously. Therefore, the grouping and prediction of aroma compounds contributes to the understanding of the anabolic metabolism, as well as offers insight into the sensory differences [14].

According to chemical classes, aroma compounds were grouped and built RF regression models, meanwhile compared different pre-treatment method for HSI of apple, presented in Table 1. The modeling results showed that predictions were good in all chemical classes. Overall, these spectral pre-treatments gave similar results in all chemical classes, SNV resulting slightly better. SS and D1 were not stable enough and sometimes even reduced the accuracy of predictions. SNV also performed well in previous spectroscopic studies of apple [25,26].

**Figure 3:**
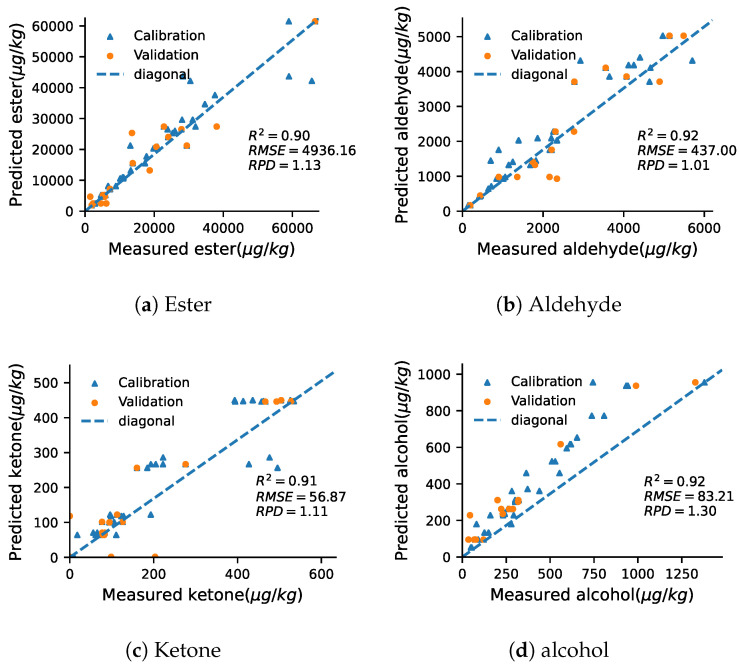
Scatter plots of measured and predicted Ester (**a**), Aldehyde (**b**), Ketone (**c**) and Alcohol (**d**).

**Figure 4:**
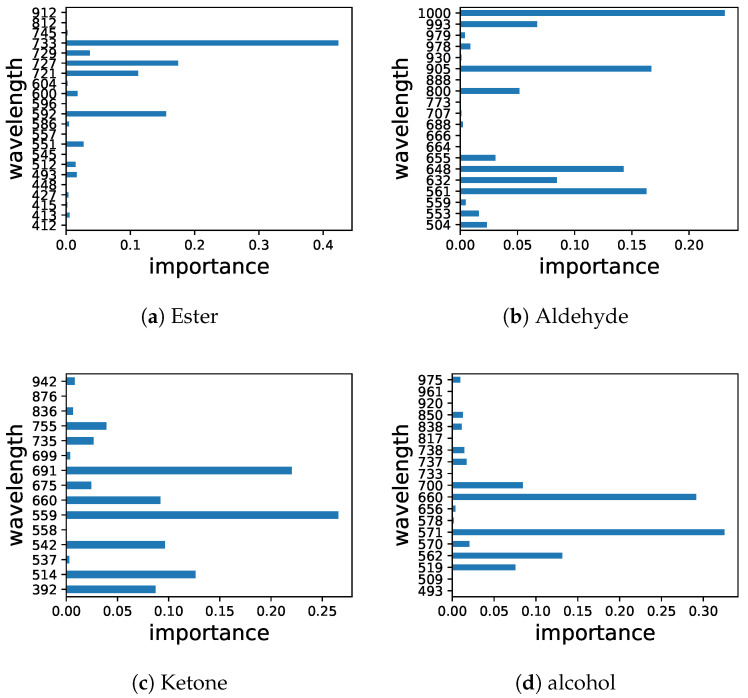
Distribution of characteristic spectra band Ester (**a**), Aldehyde (**b**), Ketone (**c**) and Alcohol (**d**).

The prediction accuracy of ester and alcohol was highest among all chemical groups, showing $R^{2}$ values above 0.9 for calibration and cross-validation dataset. In addition, $R^{2}$ values of aldehydes and ketones for cross-validation dataset was approximately 0.85, $R^{2}$ values for calibration dataset was similar to that of alcohol, thus might be useful. An additional model stability metric was RPD. As shown in Table 1, the RPD values for all SNVs range from 0.9–1.45. Generally, RPD values between 1.4 and 2 could be considered as fairly reliable, for quantification, values above 2 are desired [27]. Compared to other fruit, the $R^{2}$ of this work is better than coffee [14], even if the best effect of alcohol, but worse than grape [16]. However, the modeling effect of this work is similar to coffee. On conclusion, although SNV was acceptable from $R^{2}$, the RF model was still not stable enough from RPD. Since SNV had a strong correlation occurred in RF model for some chemical groups, measured values were plotted against predicted values in Figure 3. According to the results of the grid search algorithm, the corresponding characteristic wavelengths of the chemical groups were counted also shown in Figure 4.

As mentioned earlier, the band range of visible light about 400–750 nm. Although all the characteristic spectral ranges of the chemical group models exceed the range of visible light, the characteristic spectra of ester and alcohol played a major role almost are in the visible light range, while the most important characteristic band of aldehyde is 1000 nm, and ketone also have some of the more important spectra distributed outside the visible light range. The characteristic wavelengths of ketones were also described in grape.However, in this work, not only does the number of characteristic wavelength increase significantly, but the interval shifts toward region of the infrared obviously [16].

### 3.4. HSI Prediction of Single Volatile Compounds of Apple

Some volatile compounds individually, as shown in Table A1. The coefficient of determination $R^{2}$ and the root mean square error (RMSE) for calibration and cross-validation datasets were included in the result, to assess the reliability of models.

Although the prediction of ester and alcohol was reliable, the result of single compounds of ester and alcohols was unstable in both of calibration and cross-validation dataset, with $R^{2}$ less than 0.7, except for Cyclooctanol. With 0.7 as the reliability threshold, only Cyclooctanol, Undecenal meet the requirements among all chemical compounds, and the $R^{2}$ value of other chemicals almost between 0.3 and 0.6, which means their performance was not good. Therefore hyperspectral can predict a single compound, but unfortunately Cyclooctanol and Undecenal, which were predicted effectively in the RF model, were not the main aroma components of apple.

### 3.5. Visualization of Apple Aroma Compounds

**Figure 5:**
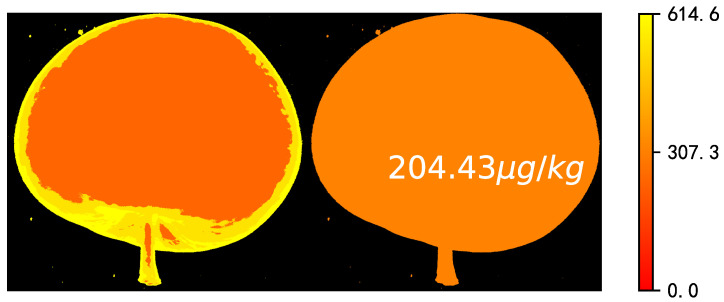
Pixel-wise (**left**) and object-wise (**right**) visualization of alcohol.

Visualization alcohol were generated in pixel-wise and object-wise manners using the developed RF model and preprocessed spectra, and prediction values expressed by a linear color bar (legend). The pixel-wise prediction maps could provide spatial distribution of the alcohol, while the object-wise maps provided reliable evidence for apple grading by visualization of overall quality traits, related with the average spectra for the whole fruit. As shown in Figure 5, a variation of alcohol within the fruit could be observed in which prediction values were lower in the central area with more pixels in red, but the peripheral area with more pixels in yellow was higher. The variation might be caused by differences in sunlight exposure during growth, and the same distribution was also present in SSC of apples [28]. Furthermore, Xuan Guantao once visualized the SSC of peach based on hyperspectra, and the distribution law was consistent with alcohol in this work, but its visualization effect was better [29]. The prediction average alcohol was 204.43 μg/kg, which was in close agreement with its reference value. Despite that, the transition zone of the mapping results didn’t change smoothly, and the difference within the center and surrounding areas was not obvious, which indicated the reliability of the model was insufficient. However, the mapping results still demonstrated a significant spatial approximately distribution and prediction of quality, showing the superiority of hyperspectral imaging.

## 4. Conclusions

In the breeding engineering, the rapid and non-destructive testing of aroma components of hybrid offspring can simplify the screening process and significantly reduce costs. In this study, hyperspectral imaging was employed to try to evaluate the aroma components of ‘Honeycrisp’ × ‘Maodi’ hybrid offspring. Spectra-based SNV-RF model performed well in chemical classes predictions, but didn’t well in predictions of individual chemicals. In prediction of chemical classes, result of alcohol was most reliable, followed by ester. Analysis of the characteristic spectra of chemical groups, the characteristic spectrum of ester are 413, 493, 512, 551, 592, 600, 721, 727, 729, 733 nm, while the characteristic spectrum of alcohol are 519, 562, 570, 571, 660, 676, 700, 737, 738 nm, all in the visible light range. The number of characteristic spectra of aldehyde and ketone is relatively large, respectively 20 and 19.

In prediction of individual volatile compounds, results of most chemical components prediction were poor, predictions of Cyclooctanol and 2-Undecenal alone were barely usable. To improve the methodologies studies in this work, more hybrid offspring, modeling methods and indicators will be considered and practiced in future. The result indicated the possibility of hyperspectral technology in the detection of aromatic chemical classes and individual aroma components in hybrid offspring, but the stability and reliability of the model needed to be improved, which provided a new choice for the preliminary screening of aroma characteristics in the process of apple breeding, and also provided a theoretical basis for automatic grading based on apple aroma.

## Figures and Tables

**Table 1:** Results of RF prediction of volatile compounds as chemical groups, using different spectral pre-treatment, on HSI spectra acquired on apples.

Group	Range	Method	$R_{c}^{2}$	RMSEC	$R_{v}^{2}$	RMSEP	RPD
Ester	1286.91–15,432.56	raw	0.84	7042.37	0.81	7273.12	1.04
		SNV	0.9	4936.16	0.9	4759.18	1.13
		SS	0.83	6513.77	0.83	6678.67	1.12
		D1	0.82	6562.55	0.86	5996.54	1.43
Aldehyde	173.85–12,635.05	raw	0.79	696.53	0.75	805.28	1.28
		SNV	0.92	437	0.84	649.23	1.01
		SS	0.88	517.1	0.81	702.78	1.25
		D1	0.83	648.6	0.82	615.31	1.11
Ketone	13.02–655.99	raw	0.88	57.04	0.83	75.17	1.10
		SNV	0.91	56.87	0.86	59.52	1.11
		SS	0.82	73.98	0.8	74.88	1.16
		D1	0.83	71.57	0.86	60.75	1.13
Alcohol	33.77–1504.67	raw	0.89	115.95	0.84	99.6	0.93
		SNV	0.92	83.21	0.91	112.09	1.30
		SS	0.87	103.1	0.86	100.28	0.93
		D1	0.83	125.17	0.76	148.38	1.14

Note: *R*^2^ = coefficient of determination; RMSE = root mean square error; RPD = ratio to performance deviation.

## Data Availability

Data is contained within the article.

## Appendix A

**Table A1:** Performance of RF regression model to predict individual volatile compounds by HSI scanning of apples.

Group	Name	Range	$R_{c}^{2}$	RMSEC	$R_{v}^{2}$	RMSEP
Ester	Ethyl acetate	0–476.48	0.26	64.54	0.35	51.35
	Caprylic acid N-butyl ester	0–2624.29	0.45	489.28	0.38	492.77
	Isoamyl caprylate	0–2089.09	0.27	257.51	0.24	326.93
	Ethyl caprylate	0–357.39	0.35	63.55	0.32	78.58
	amyl octanoate	0–558.61	0.38	87.46	0.33	85.19
	Hexyl octanoate	0–1713.13	0.35	88.13	0.22	145.53
	Propyl octanoate	0–271.70	0.42	52.05	0.27	58.62
	Ethyl hexanoate	0–549.01	0.3	79.62	0.49	66.87
	Amyl caproate	0–7495.30	0.4	445.13	0.23	608.85
	Hexyl hexanoate	0–47,995.00	0.34	3110.29	0.29	2881.89
	Caproic acid propyl ester grade I	0–750.94	0.54	120.2	0.5	108.79
	Heptanoic acid, butylester	0–361.20	0.55	54.07	0.48	51.93
	Heptanoic acid isoamyl ester	0–252.41	0.37	22.74	0.36	28.75
	Butanoic acid, 3-methylbutyl ester	0–860.87	0.48	83.68	0.43	79.37
	Ethyl butyrate	0–746.58	0.39	99.28	0.21	126.48
	Amyl butyrate	0–1126.70	0.49	64.79	0.32	79.24
	n-butyl butyrate	0–2600.97	0.38	425.38	0.25	401.32
	n-Propyl propionate	0–159.70	0.39	12.69	0.22	18.85
	Isoamyl propionate	0–166.31	0.44	25.84	0.36	33.59
	Butyl Propionate	0–1810.80	0.47	213.35	0.3	251.02
	2-Methyl Butyl Propionate	0–570.28	0.43	93.13	0.28	107.16
	Hexyl benzoate	0–116.48	0.48	17.15	0.32	16.42
	Butyl 2-methylbutyrate	0–12,156.00	0.28	1342.21	0.34	1216.25
	Butanoic acid,2-methyl-, 1-methylethyl ester	0–464.43	0.29	86.04	0.23	96.6
	2-methylbutyl acetate	47.22–10,913.10	0.36	790.66	0.37	693.59
	2-Pentanol propanoate	0–308.83	0.22	36.15	0.31	30.45
Aldehyde	Octanal	0–634.46	0.28	65.17	0.21	54.22
	Hexanal	6.98–7989.12	0.37	873.76	0.24	1068.92
	Nonanal	0–990.50	0.32	157.76	0.34	172.58
	Decanal	0–881.03	0.4	37.45	0.36	51.24
	Undecenal	0–393.94	0.99	0.13	0.78	0.41
	trans-2-Pentenal	0–78.03	0.42	14.22	0.35	13.84
	trans-2-Nonenal	0–385.19	0.39	36.7	0.31	38.82
	(E)-2-Octenal	0–391.01	0.52	31.67	0.3	45.68
	5-Hydroxymethylfurfural	0–18,504.71	0.39	525.55	0.31	506.01
	(E)-2-tridecen-1-al	0–24.19	0.8	2.19	0.6	2.44
	2-methylpent-4-enal	0–857.36	0.5	107.39	0.35	85.57
	Heptenal	0–796.97	0.32	126.53	0.27	116.0
Ketone	6-Methylhept-5-en-2-one	0–432.67	0.41	79.07	0.21	97.56
	6,10-Dimethyl-5,9-undecadien-2-one	0–176.85	0.34	23.95	0.35	17.8
	1-Octen-3-one	0–407.27	0.47	49.98	0.37	64.18
	1-Penten-3-one	0–118.58	0.42	19.68	0.39	22.66
Alcohol	1-Octanol	0–88.82	0.42	13.75	0.24	14.47
	Ethanol	0–420.63	0.24	68.25	0.26	62.02
	Furfuryl alcohol	0–2690.80	0.75	72.58	0.46	105.08
	cyclooctanol	0–170.87	0.94	6.05	0.9	8.58
	trans-2-Hexen-1-ol	0–191.16	0.36	42.9	0.23	40.01
	2-Methylbutan-1-ol	0–3622.93	0.44	152.12	0.31	217.03

## References

[1] Khan M.A., Olsen K.M., Sovero V., Kushad M.M., Korban S.S. (2014). Fruit Quality Traits Have Played Critical Roles in Domestication of the Apple. Plant Genome.

[2] Nardini G.S., Merib J.O., Dias A.N., Dutra J.N., Silveira C.D., Budziak D., Carasek E. (2013). Determination of volatile profile of citrus fruit by HS-SPME/GC-MS with oxidized NiTi fibers using two temperatures in the same extraction procedure. Microchem. J..

[3] Lu Y., Saeys W., Kim M., Peng Y., Lu R. (2020). Hyperspectral imaging technology for quality and safety evaluation of horticultural products: A review and celebration of the past 20-year progress. Postharvest Biol. Technol..

[4] Siche R., Vejarano R., Aredo V., Velasquez L., Saldana E., Quevedo R. (2016). Evaluation of Food Quality and Safety with Hyperspectral Imaging (HSI). Food Eng. Rev..

[5] Mollazade K., Omid M., Tab F.A., Mohtasebi S.S. (2012). Principles and Applications of Light Backscattering Imaging in Quality Evaluation of Agro-food Products: A Review. Food Bioprocess Technol..

[6] Mendoza F., Lu R., Ariana D., Cen H., Bailey B. (2011). Integrated spectral and image analysis of hyperspectral scattering data for prediction of apple fruit firmness and soluble solids content. Postharvest Biol. Technol..

[7] Ding C., Feng Z., Wang D., Cui D., Li W. (2021). Acoustic vibration technology: Toward a promising fruit quality detection method. Compr. Rev. Food Sci. Food Saf..

[8] Feng Z. (2017). Clamping Device for Detection of Moldy Core in Apples. Master’s Thesis.

[9] Ma T., Xia Y., Inagaki T., Tsuchikawa S. (2021). Non-destructive and fast method of mapping the distribution of the soluble solids content and pH in kiwifruit using object rotation near-infrared hyperspectral imaging approach. Postharvest Biol. Technol..

[10] Chu X., Miao P., Zhang K., Wei H., Fu H., Liu H., Ma Z. (2022). Green Banana Maturity Classification and Quality Evaluation Using Hyperspectral Imaging. Agriculture.

[11] Fan Z., Wang D., Zhang N., Zhou B. (2022). Monitoring of Nitrogen Transport in Pear Trees Based on Ground Hyperspectral Remote Sensing and Digital Image Information. J. Chem..

[12] Chen X., Yi H., Wang N., Zhang M., Jiang S., Xu J., Mao Z., Zhang Z., Wang Z., Jiang Z. (2022). The selection of bud varieties promotes the high-quality and efficient development of the world’s apple and citrus industries. Chin. Agric. Sci..

[13] Klee H.J. (2010). Improving the flavor of fresh fruits: Genomics, biochemistry, and biotechnology. New Phytol..

[14] Su W.H., Sun D.W., He J.G., Zhang L.B. (2018). Variation analysis in spectral indices of volatile chlorpyrifos and non-volatile imidacloprid in jujube (Ziziphus jujuba Mill.) using near-infrared hyperspectral imaging (NIR-HSI) and gas chromatograph-mass spectrometry (GC–MS). Food Res. Int..

[15] Caporaso N., Whitworth M.B., Cui C., Fisk I.D. (2017). Variability of single bean coffee volatile compounds of Arabica and robusta roasted coffees analysed by SPME-GC-MS. Comput. Electron. Agric..

[16] Jin X., Liu L., Liu Y., Guo Y., Mao D. (2020). Hyperspectral imaging technology for non-destructive testing of grape aroma components at different ripening stages. Food Ind..

[17] Aprea E., Gika H., Carlin S., Theodoridis G., Vrhovsek U., Mattivi F. (2011). Metabolite profiling on apple volatile content based on solid phase microextraction and gas-chromatography time of flight mass spectrometry. J. Chromatogr. A.

[18] Yan D., Shi J., Ren X., Tao Y., Ma F., Li R., Liu C. (2020). Insights into the aroma profiles and characteristic aroma of ‘Honeycrisp’ apple (Malus × domestica). Food Chem..

[19] Wu X., Bi J., Fauconnier M.L. (2022). Characteristic Volatiles and Cultivar Classification in 35 Apple Varieties: A Case Study of Two Harvest Years. Foods.

[20] Ci Z., Tian L., Liu X., Mou H., Song L. (2022). Research progress on the aromatic substance of Fuji apple. Chin. Fruit Trees.

[21] Shao Y., Wang K., Xuan G., Gao C., Hu Z. (2021). Soluble solids content monitoring for shelf-life assessment of table grapes coated with chitosan using hyperspectral imaging. Infrared Phys. Technol..

[22] Mo C., Kim M.S., Kim G., Lim J., Delwiche S.R., Chao K., Cho B.K. (2017). Spatial assessment of soluble solid contents on apple slices using hyperspectral imaging. Biosyst. Eng..

[23] Ignat T., Lurie S., Nyasordzi J., Ostrovsky V., Egozi H., Hoffman A., Schmilovitch Z.E. (2014). Forecast of Apple Internal Quality Indices at Harvest and During Storage by VIS-NIR Spectroscopy. Food Bioprocess Technol..

[24] Yuan X. (2021). Research on Nondestructive Detection System of Apple Suger Content Based on Hyperspectral Imaging Technology. Master’s Thesis.

[25] Keresztes J.C., Goodarzi M., Saeys W. (2016). Real-time pixel based early apple bruise detection using short wave infrared hyperspectral imaging in combination with calibration and glare correction techniques. J. Spectrosc..

[26] Zhu X., Li G. (2019). Rapid detection and visualization of slight bruise on apples using hyperspectral imaging. Int. J. Food Prop..

[27] Ncama K., Opara U.L., Tesfay S.Z., Fawole O.A., Magwaza L.S. (2017). Application of Vis/NIR spectroscopy for predicting sweetness and flavour parameters of ‘Valencia’ orange (*Citrus sinensis*) and ‘Star Ruby’ grapefruit (*Citrus* x *paradisi* Macfad). J. Food Eng..

[28] Jiang X., Zhu M., Yao J., Zhang Y., Liu Y. (2022). Study on the Effect of Apple Size Difference on Soluble Solids Content Model Based on Near-Infrared (NIR) Spectroscopy. J. Spectrosc..

[29] Xuan G., Gao C., Shao Y. (2022). Spectral and image analysis of hyperspectral data for internal and external quality assessment of peach fruit. Spectrochim. Acta. Part A Mol. Biomol. Spectrosc..

